# Exercise oximetry in clinical practice: A single‐centre perspective on procedure and techniques

**DOI:** 10.1113/EP092711

**Published:** 2025-05-11

**Authors:** Simon Lecoq, Jeanne Hersant, Mathieu Feuilloy, Nafi Ouedraogo, Mariève Houle, Pierre Abraham

**Affiliations:** ^1^ Service of Sports Medicine University Hospital Angers France; ^2^ Service of Vascular Medicine University Hospital Angers France; ^3^ INSERM, CNRS, MITOVASC, Equipe CarMe, SFR ICAT University Angers Angers France; ^4^ School of Electronics (ESEO) Angers France; ^5^ UMR CNRS 6613 LAUM Le Mans France; ^6^ Institut Supérieur des Sciences de la Santé Université Nazi BONI Bobo‐Dioulasso Burkina Faso; ^7^ Department of Anatomy Université du Québec à Trois‐Rivières Québec Canada

**Keywords:** claudication, diagnosis, exertional limb pain, oxygen, peripheral artery disease, treadmill testing

## Abstract

In moderate lower extremity artery disease (LEAD), when tissue ischaemia due to impaired inflow occurs at exercise but not during rest, exercise oximetry may be evaluated as a part of the diagnosis process. Initially used when assessing critical limb ischaemia at rest, transcutaneous oximetry (TcpO_2_) has also been used in the last two decades during exercise assessment as a non‐invasive method to measure oxygen pressure at the skin's surface, offering insights into loco‐regional oxygen delivery–requirement mismatch. The introduction of decrease from rest of oxygen pressure (DROP) analysis in the TcpO_2_ technique, which corresponds to the difference between limb oxygen pressure changes and chest oxygen pressure changes from rest, provides new information about the severity of the local ischaemia during exercise. In this paper, we elucidate the utilization of TcpO_2_ during exercises (Ex‐TcpO_2_) over the years and provide information about how the technique has evolved and how the changes in the testing procedures have provided the opportunity for detecting abnormalities in both vascular and non‐vascular clinical practice. We discuss the importance of Ex‐TcpO_2_ in the diagnosis of peripheral artery disease and its valuable contribution as a differential diagnostic tool for patients with co‐morbid conditions such as lumbar spinal stenosis. We also provide recommendations about the utilization of Ex‐TcpO_2_ and contribute to a better understanding of the techniques in terms of efficacy, limitations and clinical applications. However, clarifications about its role in the diagnostic algorithm are needed, to ensure a better integration of the technique in clinical practice.

## Abstract (highlights)

- **What is the topic of this review?**
  This study review describes the technique of exercise transcutaneous oximetry and provide recommendation on its utilization.
- **What advances does it highlight?**
  This review highlights the importance of the technique for both vascular and non‐vascular medical problems.

## 1 INTRODUCTION

In moderate lower extremity artery disease (LEAD), before ischaemia at rest occurs, impaired inflow occurs during exercise and results in tissue ischaemia. Ischaemia is characterized by insufficient oxygen delivery, due to impaired arterial inflow, to meet increased tissue oxidative metabolic requirements from rest. Transcutaneous oximetry (TcpO_2_) was proposed in medicine about 50 years ago to estimate the imbalance in oxygen delivery (Peabody et al., 1978; Riegel & Versmold, 1979). The technique measures local oxygen pressure at the skin's surface, offering the advantage of measuring the oxygen delivery–requirement mismatch rather than only arterial blood pressure or arterial inflow, but also of systemic (chest) oxygen pressure ($P_{O_{2}}$) changes. Its major limitation is the unpredictable transcutaneous oxygen pressure gradient between deep tissues and the skin surface. First attempts to use TcpO_2_ during exercise (Ex‐TcpO_2_) date back to the eighties (Caillard et al., 1990; Hauser & Shoemaker, 1983; Modesti et al., 1990; Provan & Ameli, 1986). Most authors accounted for potential systemic changes by expressing limb TcpO_2_ results as a regional perfusion index (RPI; ratio of limb values and chest values) (Hauser & Shoemaker, 1983). A 2003 publication in *Circulation* proposed to analyse the results through the ‘decrease from rest of oxygen pressure’ (DROP) instead of RPI calculation (Abraham et al., 2003; Bouye et al., 2004), renewing interest in Ex‐TcpO_2_, because DROP is completely independent of the (unpredictable) transcutaneous gradient.

Ex‐TcpO_2_ has been used routinely in a few referral services in the last decades (Abraham et al., 2020, 2021, 2005, 2003; Abraham, Colas‐Ribas, et al., 2018; Dinesh et al., 2023; Leenstra et al., 2021; Mahe et al., 2020; Manunga et al., 2023; Mirza et al., 2020). Since the beginning of our practice, a database has been established to record the characteristics of patients and the results of the investigations. As a longitudinal recording (with retrospective analysis) of routine clinical procedures and according to French law, patients were aware that they could refuse the recording of their results in the database, which was administratively approved, under reference no. CNIL 2017‐002, without the need for written consent. This database, to date, contains the results of more than 9000 tests. The present paper aims to explain why and how the technique has been used by extracting all data from the beginning of the database until April 2023 (*n* = 8995). We focus on what modifications occurred in our routine practice and what we have observed in our database and learned from our practice in the last two decades. We believe that this review can contribute to a better understanding of the interests and limits of Ex‐TcpO_2_.

## 2 POPULATION AND METHOD GENERAL DESCRIPTION

### 2.1 Description of the population

The population included in this narrative review was 18.8% females and 81.2% males. The mean weight of females and males was 66.40 ± 15.13 and 79.39 ± 14.67 kg, respectively. The mean height of females and males was 159.18 ± 6.52 cm and 170.89 ± 6.95 cm, respectively. The mean age of all individuals was 63.87 ± 11.51 years. Regarding medication intake, 6825 individuals reported taking antiplatelet medication, 5249 reported taking medication for high blood pressure, 5673 reported taking lipid‐lowering medication, 658 reported taking anticoagulants, 2643 reported taking beta‐blockers, and 2127 reported taking medication for diabetes. In addition, 4369 individuals were smokers.

### 2.2 Description of the method

TcpO_2_ is a non‐invasive measure of the partial pressure of oxygen that diffuses from sub‐cutaneous tissue to the skin surface. A probe holder is fixed to the skin with a double‐sided adhesive. The cup of the holder is then partly filled with a contact liquid to remove air bubbles. A video is accessible in one of our previous publications to describe precisely how probes are fixed and secured on the skin (https://ars.els‐cdn.com/content/image/1‐s2.0‐S0021915018312206‐mmc1.mp4).

**FIGURE 1:**
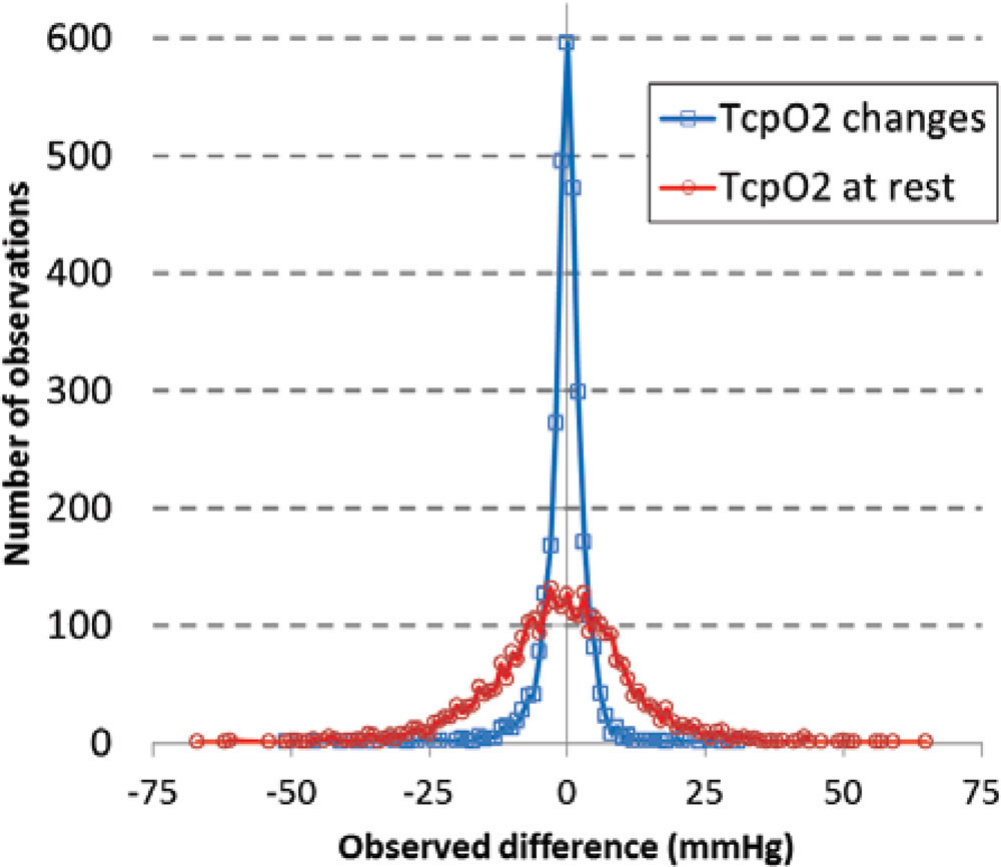
Results of the absolute TcpO_2_ difference at rest and the difference in absolute maximal decrease. Results of the absolute TcpO_2_ difference at rest (red circles) and the difference in absolute maximal decrease (blue squares) from 3221 assessment with simultaneous right and left chest probes recording during Ex‐TcpO_2_. The absolute maximal decrease refers to the largest decrease in oxygen pressure measured via Ex‐TcpO_2_ during or after the exercise test. As shown, the variability of absolute changes is much smaller than the difference between simultaneous recordings from the two chest probes, confirming the high reliability of TcpO_2_ changes despite large differences in the absolute starting values between the probes.

Once in place, users must allow a period of 10–15 minutes of local heating to achieve maximal vasodilatation and relatively stable TcpO_2_ values. Next, the system measures the $P_{O_{2}}$ at the predefined temperature (we use 44.5°C) and provides a value (mmHg) that is mathematically corrected to be expressed for a value of 37°C. Despite local heating, the difference between surface $P_{O_{2}}$ and underlying tissue $P_{O_{2}}$ is unpredictable and depends on factors such as capillary density, subcutaneous fat and local oxygen consumption. As per our routine procedure, when eight probes are available, we usually record calves, thighs, buttocks and chest on both sides. It could be suggested that two chest probes are not needed since chest changes are similar on both sides. While this is generally the case, there may be some exceptions, possibly because of unknown sub‐clavicular artery stenosis, electronic drift or probe disconnection. Furthermore, since all peripheral lower limb values use the chest as a reference to calculate DROP, in the event of a chest disconnection, the other probe can be used to replace the disconnected one. Chest probe disconnection is fairly rare, and has occurred in only 0.4% of the assessments conducted to date. As shown in Figure 1, when two probes were used on the chest in our patients, chest between‐probe difference in TcpO_2_ at rest shows a standard deviation (SD) of 13.7 mmHg, suggesting that differences in absolute value up to 27 mmHg (2 SD) between two chest probes are to be considered within the normal range. As illustrated in the figure and since absolute changes over time for two probes are highly correlated regardless of their respective starting value (Henni, Sempore, et al., 2018), the SD of absolute changes is much smaller (4.8 mmHg).

These points are of major clinical importance for two reasons. First, they explain why the RPI was not very reliable, as the expected variability of a ratio is twice the variability of its numerator and denominator (Bouye et al., 2004). Second, this explains why the exact positioning of the chest reference probe is of little, if any, interest in routine measurements because it has little, if any, effect on DROP calculation, as DROP is independent of starting values.

**FIGURE 2:**
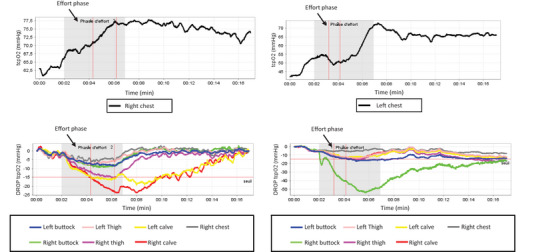
Examples of data collection using TcpO_2_ from two different patients. As shown in these examples, DROP values (lower graphs) return to zero at end of recovery, while the chest absolute value is much higher than its starting value. All DROP values are calculated using the left chest electrode, and the DROP for the right chest electrode (thorax droit) remains stable throughout the exercise (effort phase: grey area) and recovery period, confirming that both chest changes were almost similar.

All tests begin with a resting period of at least 30 s. The average absolute value of each probe over the resting period (*r*) will then be used for DROP calculation. The DROP is calculated at all times (*t*), by subtracting chest TcpO_2_ changes (Chest_t_ − Chest_r_) from limb TcpO_2_ changes (Limb_t_ − Limb_r_). By construction, the average DROP is zero over the resting period. The software used to interpret the DROP in real time is in‐home software that only calculates the DROP values. In the absence of limb ischaemia in patients, as well as in normal subjects, changes at the limb level will be similar to changes at the chest level, and DROP will remain close to zero throughout the walking and recovery period. In the presence of regional blood flow impairment, DROP should decrease in proportion to the severity of local ischaemia during the walking period. Additionally, DROP could sometimes decrease further during the first seconds or minutes following the walking period. This could be explained by a possible decrease in the chest reference value during exercise, which may be related to systemic hypoxaemia (Abraham, Gu, et al., 2018) or by the need of covering oxygen dept with persistent increased oxygen consummption after the end of exercise. The lowest DROP value (DROPm) observed during the walking and recovery periods is used for the interpretation. A recovery of 10 minutes was sufficient in more than 90% of our tests for DROP to return to stable values close to zero. In theory, DROP at the end of the recovery period should be exactly zero even if the absolute (systemic and limb) values are generally much higher after 10 min of recovery than their respective starting values (see example in Figure 2).

Although, in most cases, this is true, a technical issue may slightly interfere with this expected result. Once calibrated, the recorded value of TcpO_2_ under stable condition (such as in a gas chamber) may show a slow drift along time even after careful calibration. What can be done, is to correct for this drift assuming that it is almost constant over time.

## 3 CHANGES THAT OCCURRED OVER TIME

### 3.1 Changes of walking procedure

**FIGURE 3:**
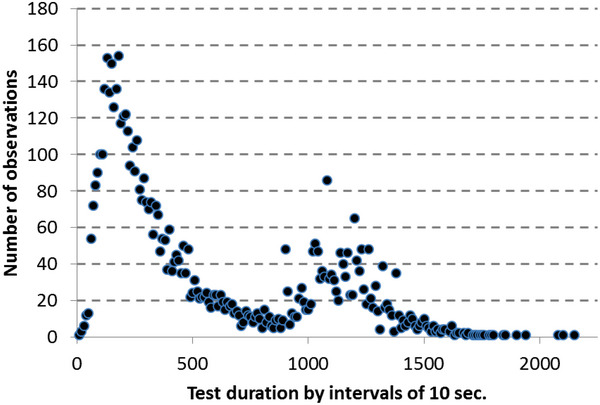
Distribution of the durations of tests using the biphasic procedure used since 2010. The results reported here are for 5833 biphasic procedures (constant 3.2 km/h and 10% slope for the first 15 min followed by an incremental speed and slope procedure for patients that are not limited during the first phase).

At the beginning of our practice, we performed a 3.2 km/h (2 mph) with a 10% slope constant load protocol for all patients, maximizing the duration of the test to 20 minutes. Rapidly, we began measuring spontaneous walking speed over a 10 meters distance, and an alternative protocol was proposed for all slow walkers identified (patients who take more than 15 seconds to walk 10 meters). This alternative protocol was conducted with a maximal speed of 2 km/h (Sempore et al., 2020). Inversely, we found that 16.4% of the patients reached the 20 minutes time point of the 3.2 km/h test with or without symptoms resulting in a ceiling effect of the protocol. Then, from 2010, we changed our procedure to a biphasic protocol, which means that after 15 minutes of walking at 3.2 km/h with a 10% slope with an absence of limitation, we increased both speed and slope by steps of 1 minute to avoid the ceiling effect. Using this procedure, all patients were symptom limited, and 73.6% of patients stopped before the incremental phase of the procedure with a log‐normal distribution of their maximal walking distance (Figure 3).

### 3.2 Changes of devices

We initially used five single‐probe TCM5 (Radiometer Brønshøj, DK) devices connected to an analog/digital card converter, which was eventually upgraded over time to the six‐probe Radiometer TCM400 and finally to the eight‐probes Perimed PF6000 (Perimed, Järfälla, Sw). Both systems (Perimed and Radiometer) use the same Clark‐type electrodes. These electrodes consist of a platinum cathode and a silver anode. During measurement, the O_2_ diffuses through a membrane into the electrode, where the O_2_ is reduced, generating a measurable current. The intensity of the current determines the transcutaneous partial pressure of oxygen. Other photo‐reactive systems exist, in which the light from a green light‐emitting diode (LE) excites the sensor spots in the matrix layer to emit fluorescence (Dasgupta et al., 1993). If the sensor spot encounters an oxygen molecule, the excess energy is transferred in a non‐radiative way, quenching the fluorescence signal. The degree of quenching correlates to the partial pressure of oxygen in the manufacturer's matrix, which is in dynamic equilibrium with oxygen in the sample. The decay time measurement is internally referenced and provides $P_{O_{2}}$ values in millimetres of mercury. In theory, these photo‐reactive systems are advantageous because they do not consume oxygen locally, but in practice, their response to ischaemia seems to be slow (Urban et al., 2015).

### 3.3 Changes of operators that performed the technique

For about 8 years, all tests were performed by a single operator (P.A.). Thereafter, the number of operators progressively increased, with an average of 10 different operators trained each year in recent years (median total number of tests per operator: 66). To date, more than 60 operators have been trained in our laboratory. Based on our single‐centre experience, the minimal number required for training is at least 20 tests.

### 3.4 Changes of referring physicians

In parallel with the information about the availability of the technique and presentation of the initial results, physicians that referred patients were initially only surgeons and vascular medicine physicians from our hospital. We progressively received over time patients referred by other specialists or other physicians, such as private practice vascular physicians and extended to an orthopaedic surgeon, rheumatologists and family physicians. These changes have been accompanied by an extension of the indications to include mainly the follow‐up of walking tolerance under treatment, the diagnosis of atypical claudication, and the differential diagnosis of lumber spinal stenosis.

### 3.5 Changes of the population studied

**FIGURE 4:**
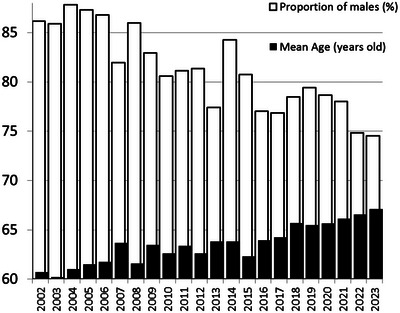
Evolution of age and sex of referred patients in the last 22 years. Results reported here are for 8936 observations out of 8995, due to some cases of age or sex being missing from the encoding.

The first change that we observed in the database was a progressive increase in the mean age of referred patients, which increased by almost 7 years over 20 years of practice (Figure 4). An optimistic explanation is that a better treatment of cardiovascular risk factors has delayed the appearance of arterial lesions or the mean age at which symptoms occur in peripheral artery disease (PAD) patients. Another view may be that we nowadays treat more patients at older ages with angioplasty than we did at a time when the bypass was the principal or only revascularization option (Harris et al., 1996). Less optimistic views include the idea that an increased sedentary lifestyle in the population results in patients remaining asymptomatic longer, or that the delays to obtain a walking test have increased due to our inability to cover the increasing demand of the number of tests. Another obvious, although puzzling, observation is the change in the sex ratio of the subjects that we tested. Whereas a male dominance remains present, an increasing proportion of females have been investigated every year. Since cardiovascular risk in females increase abruptly after menopause, it is possible that the sex ratio change resulted solely from the progressive drift in the mean age of the tested patients. It is also possible that it results from a better awareness of cardiovascular disease in women observed in the last decades (Mosca et al., 2013).

## 4 INTEREST IN EX‐TcpO_2_ IN VASCULAR AND NON‐VASCULAR MEDICINE

One principal interest of Ex‐TcpO_2_ is in the evaluation of proximal claudication, which can result from aorto‐iliac lesions. As underlined by the Society for Vascular Surgery (Society for Vascular Surgery Lower Extremity Guidelines Writing et al., 2015), iliac disease is much harder to diagnose than other lower limb lesions. As we previously reported, the annual increase in the number of Ex‐TcpO_2_ tests has resulted in a parallel increase in the number of internal iliac revascularizations in our service (Abraham, Colas‐Ribas, et al., 2018). This ability to detect proximal exercise‐induced ischaemia is also of specific interest in patients suspected of lumbar spine stenosis (Gahier et al., 2020). Indeed, PAD patients may suffer from numerous co‐morbid conditions including chronic cardiac, pulmonary or osteoarticular diseases. As for lumbar spine stenosis, there is no parallel between the radiological severity of spine lesions and walking disability, and symptoms of arterial and neural claudication may be confusing (Kauppila, 1997, 2009; Kauppila & Tallroth, 1993; Kauppila et al., 2004, 1994). Clearly, due to the limited number of centres performing these tests, not all patients with suspected lumbar spine stenosis can be referred for Ex‐TcpO_2_. We proposed a simple index to estimate the probability that Ex‐TcpO_2_ might be positive in such patients (Gahier et al., 2020). For pulmonary diseases, we showed that Ex‐TcpO_2_ allows the analysis of systemic arterial $P_{O_{2}}$ changes at exercise (through chest TcpO_2_ changes) and detects a significant amount of unknown pulmonary diseases in patients referred for claudication (Abraham, Gu, et al., 2018; Colas‐Ribas et al., 2016). The arterial $P_{O_{2}}$ (and then chest TcpO_2_) in normal patients is expected to slightly increase during exercise (due to improved ventilation/perfusion ratio during moderate exercise) (Ouedraogo et al., 2011). A decrease during exercise can be used as a surrogate marker of exercise‐induced hypoxaemia (Bruneau et al., 2013; Colas‐Ribas et al., 2016). In the ageing population, chronic airway diseases and PAD share similar risk factors. Additionally, the possibility of performing recordings in specific regional areas may be of interest. Nowadays, 20% of the patients are referred to us by non‐vascular specialists, including spine surgeons, orthopaedic surgeons and neurosurgeons, to investigate the possibility of a vascular origin of pain (mainly as a differential diagnosis of sciatica or lumbar spine stenosis) or to differentiate between a vascular versus cardio‐respiratory origin of walking limitation.

## 5 COMPARISON WITH OTHER TECHNIQUES

The most widely used technique for evaluating the haemodynamic effects of exercise on lower limb lesions is the measurement of ankle to brachial index (ABI). It is readily accessible, cost‐effective and not technically demanding. However, post‐exercise ABI has limitations such as arterial stiffness, arrhythmia and the inability to tolerate the strict lying position after exercise. Additionally, it only accounts for lesions on the aorto‐distal axis but not on arterial branches (Jacquinandi et al., 2004; Picquet et al., 2013; Ramondou et al., 2020). A clever way of improving pressure measurements is to perform segmental recordings (toe, thigh or penile pressure indices), although penile pressure was reported to have poor sensitivity in the detection of impaired hypogastric perfusion compared to Ex‐TcpO_2_ (Mahe et al., 2009). Last, stiffness and arrhythmia should interfere with resting and post‐exercise ABI results (Hendriks et al., 2016; Henni, Ammi, et al., 2018; Laivuori et al., 2022; Lilly et al., 2013; Liu et al., 2011).

Another candidate for routine exercise testing in PAD patients is near infra‐red spectroscopy (NIRS), which claims to measure muscle saturation, although it has been reported to be highly sensitive to skin flow changes (Davis et al., 2006). This technique allows a deep penetration of muscle tissues, and it is less dependent on the external environment (Baltrūnas et al., 2022). A study by Fuglestad et al. (2020) showed that the correlation between the gastrocnemius muscle saturation and the walking distance measured with a 6‐min walking test is strong (Fuglestad et al., 2020). Nevertheless, NIRS seems to have a lower performance compared to Ex‐TcpO_2_, at least at the buttock level (Bouye et al., 2005). Furthermore, unlike TcpO_2_, which uses a chest reference probe, NIRS cannot discriminate between local ischaemia and the local effect of exercise‐induced systemic hypoxaemia. Another limitation of the use of NIRS for the diagnosis of PAD is the lack of a standardized approach for its utilization, as there is actually no consensus regarding sensor placement and acquisition parameters (Baltrūnas et al., 2022).

A comparison of Ex‐TcpO_2_ to angiography has previously been reported, and angiography was used to define the cut‐off point of the DROPm value that needs to be used for clinical routine. We use minus 15 mmHg for all our experiments at all levels (e.g., buttocks, thighs and calves) (Abraham et al., 2003, 2005).

## 6 COST, BENEFIT, ADVANTAGES AND LIMITS

Apart from the acquisition of a treadmill and electrocardiogram (ECG) system if one aims to monitor ECG in these high cardiovascular risk patients, the initial investment to specifically perform Ex‐TcpO_2_ ranges from €30,000 to €50,000 and consumables from €5 to €20 per test, depending on the number of probes used. The first large series of routine tests have emerged in Europe at the beginning of the century, and although the Mayo Clinic in Rochester (USA) started the technique in 2015 (Mahe et al., 2015; Sen et al., 2018), to our knowledge Ex‐TcpO_2_ is still not reimbursed in Europe, whereas it is now considered a refundable test in the USA. This is a frustrating situation that limits the diffusion of the technique in European countries. The Ciney‐Soft protocol was performed as a multi‐centre protocol among all national French departments that have started to use the procedure as we have. Although the medico‐economic evaluation of the study was quite simplistic, it appears that performing TcpO_2_ significantly impacts diagnostic hypotheses and might lead to non‐negligible economic benefits (Henni, Mahe, et al., 2018). The Ciney‐Soft study reported results from 603 patients. Since then, encoding of pre‐ and post‐test hypotheses and probabilities have been almost systematic locally, with currently available encoding in 2858 patients (including 245 patients from the Ciney‐Soft study). This shows that the pre‐ to post‐test diagnosis may differ in almost 30% of patients.

Furthermore, of the 2008 tests for which the suspected arterial origin remained unchanged before and after the test, the probability for this diagnosis, encoded from 1 (very unlikely) to 5 (very likely), was available in 1905 files and was changed in 1172 (61.5%) cases, in which it either increased (*n* = 947) or decreased (*n* = 225).

There are, nevertheless, limitations to the use of Ex‐TcpO_2_. First, it is a time‐consuming investigation. The walking duration averages less than 10 min, but preparation, heating period, recovery from walking and debriefing makes the total duration reach 45–60 min. Could this duration be reduced, for example, by interrupting TcpO_2_ recording immediately at the end of the walking period? Since we retrieve DROP at the end of the exercise (DROPend), we extracted DROPend and DROPm from our database and compared these two parameters both through linear regression analysis and also using a receiver operating characteristic (ROC) curve approach by dichotomizing normal and abnormal DROPm values.

In summary, among the 8995 tests conducted, 8900 had bilateral results for at least one site, with 6058 patients showing at least one DROPm value <−15 mmHg and 2784 patients showing at least one DROPend value <−15 mmHg. Therefore, ending the recording of each probe immediately at the end of the walking period would lead to missing 54.0% of positive tests. Using DROPmin <−15 mmHg as the reference for a positive result, we have 30,078 recordings with both DROPmin and DROPend values with 11,401 DROPmin values <−15 mmHg. Thereafter, it could be suggested that a different threshold could be determined for DROPend. ROC curve determination of the DROPend cut‐off point against DROPmin <−15 mmHg as a positive result provides an area of 0.916 ± 0.02 (*P *< 0.001) and suggests that using a DROPend value lower than −9 mmHg as the cut‐off value would result in an 87.6% sensitivity and 71.2% specificity as compared to DROPm. Nevertheless, DROPend <−9 mmHg has never been tested against other techniques. Since all previous validation works were performed with DROPm, we do not recommend skipping the recovery phase nor use the DROP at the end of the walking period.

There are obvious advantages to measure Ex‐TcpO_2_. It is insensitive to arterial stiffness, it can be recorded throughout the walking and recovery period, it can estimate regional blood flow impairment at different levels simultaneously providing estimation not only of the diffusion (buttocks ± thighs ± calves) but also of the severity (through the DROPm value) of the ischaemia. It can detect exercise‐induced hypoxaemia as a worsening factor of exercise intolerance (Caillard et al., 1990; Colas‐Ribas et al., 2016; Modesti et al., 1990; Mouren et al., 1998; Provan & Ameli, 1986). This latter point is of major interest since we have been repeatedly questioned about how it could be that some patients with claudication and LEAD (ABI <0.9) have no ischaemia according to Ex‐TcpO_2_. The confusion comes from the fact that the specific issue of ischaemia is not the absence of blood flow but the fact that oxygen delivery to tissues is impaired. Specifically in claudication, oxygen delivery at rest may be sufficient to cover local consumption (then TcpO_2_ remains in the normal range) but become insufficient when O_2_ requirements increase during exercise while the increase in oxygen delivery is blunted. Impaired oxygen delivery mostly results from impaired inflow, but it can also result from impaired transport capacity (anaemia) or impaired oxygen content of arterial inflow (exercise‐induced hypoxaemia) as previously discussed. Since the calculation of the DROP accounts for systemic $P_{O_{2}}$ changes, the limb DROP index will remain zero if the decrease in limb TcpO_2_ mimics the decrease in chest TcpO_2_, as both result from a systemic decrease in arterial $P_{O_{2}}$. Attention must be paid during the interpretation of Ex‐TcpO_2_ tests, not only to DROP changes at the limb level, but also to the profile of chest TcpO_2_ changes during exercise (Colas‐Ribas et al., 2016; Ouedraogo et al., 2011).

## 7 DIRECTIONS FOR THE FUTURE AND CONCLUSION

The TcpO_2_ technique is simple and can be performed by a technician or a nurse under medical supervision. It is now reported as a useful tool in the ‘European Society for Vascular Surgery (ESVS) 2024 Clinical practice guidelines on the management of asymptomatic lower limb peripheral arterial disease and intermittent claudication’ (Nordanstig et al., 2024), specifically for buttock claudication. Nevertheless, its position among other tools in the diagnostic algorithm of exertional limb pain of suspected vascular origin needs to be better defined: Which population should undergo this procedure? What are the optimal indications? How many probes should be used? Our present position to perform the test almost systematically in patients with claudication may be considered excessive, while we believe that diagnostic exercise testing in general (and specifically diagnostic exercise oximetry) remains largely underused. To date, we have failed to find clinical predictive factors to identify those approximately 30% of patients for which the TcpO_2_ tests induced a change in the initial diagnostic hypothesis, including post‐exercise ABI. Claudication is an exercise‐related symptom, and relying on resting investigation to diagnose and evaluate patients suffering lower limb exertional pain should no longer be the dominant practice. Hopefully, reimbursement of Ex‐cpO_2_ by French (and other European) healthcare systems will be considered in the future. Last, beyond diagnostic testing, less is known about the potential of Ex‐TcpO_2_ to adapt rehabilitation programmes or estimate the results of medical or surgical treatments (Maugin et al., 2011; Paumier et al., 2010; Picquet et al., 2013), and future studies are needed in these directions.

## 8 CLINICAL PERSPECTIVE

Among non‐invasive tools available in vascular medicine, transcutaneous oximetry (TcpO_2_) has been largely used in critical limb ischaemia for decades and, more recently, during diagnostic treadmill tests (Ex‐TcpO_2_). The development of this non‐invasive method to measure tissue oxygenation during exercise is quite attractive because of its capacity to detect regional blood flow impairment (specifically ischaemia at the buttock level) as well as to monitor systemic oxygen pressure changes for the detection of exercise‐induced systemic hypoxaemia. The present paper shows that the utilization of the decrease from rest of oxygen pressure (DROP) variable during Ex‐TcpO_2_ provide valuable insights into the physiological response to exertion and can guide clinical decision‐making in the management of lower extremity artery disease. The testing protocol has been modified in the last years to improve accuracy and patient experience. By tailoring the walking protocol to individual patient capabilities, clinicians can better detect exercise‐induced ischaemia and assess functional limitations. The present paper also highlights TcpO_2_’s utility in diagnosing proximal claudication and differentiating vascular from non‐vascular causes of symptoms. This capability is particularly valuable in complex cases such as lumbar spine stenosis, where symptoms may overlap with peripheral artery manifestations.

## AUTHOR CONTRIBUTIONS

Conception and study design: Pierre Abraham, Simon Lecoq, and Nafi Ouedraogo. Data acquisition: Simon Lecoq, Jeanne Hersant, Mathieu Feuilloy, Pierre Abraham, Mariève Houle, and Nafi Ouedraogo. Manuscript writing: Simon Lecoq, Jeanne Hersant, Pierre Abraham, Mariève Houle, and Nafi Ouedraogo. Nafi Ouedraogo takes responsibility that this study has been reported transparently and honestly. All authors have read and approved the final version of this manuscript and agree to be accountable for all aspects of the work in ensuring that questions related to the accuracy or integrity of any part of the work are appropriately investigated and resolved. All persons designated as authors qualify for authorship, and all those who qualify for authorship are listed.

## CONFLICT OF INTEREST

P.A. has benefited from loaning devices from Radiometer, Perimed and Medicap companies in the past. The companies had no access to any of the present manuscript part, concept writing, or discussion.
